# Robotic Portal Segmentectomy in the Right Middle Lobe After Right Upper Lobectomy

**DOI:** 10.1016/j.atssr.2024.07.015

**Published:** 2024-07-31

**Authors:** Ryusuke Sumiya, Takeshi Matsunaga, Yukio Watanabe, Mariko Fukui, Aritoshi Hattori, Kazuya Takamochi, Kenji Suzuki

**Affiliations:** 1 Department of General Thoracic Surgery, Juntendo University School of Medicine, Tokyo, Japan

## Abstract

Although segmentectomy is the standard surgical procedure for small-sized peripheral non-small cell lung cancer, reports on segmentectomy for right middle robe are rare because of the anatomical feature. We report a case of an 81-year-old woman with a history of left S4 segmentectomy, left basal segmentectomy, and right upper lobectomy for multiple primary lung cancer with a part solid nodule in S4a. Owing to the increased volume of the right middle lobe following a right upper lobectomy, a right S4 segmentectomy was performed. In patients with a large right middle lobe or dilated resulting from a previous lung resection, segmentectomy is an option for preserving the lung parenchyma.

Although segmentectomy should be the standard surgical procedure for small-sized peripheral non-small cell lung cancer according to a previous study,^1^ lobectomy or wide wedge resection is generally considered in the right middle lobe because the right middle lobe is a small proportion of the total lung.^2^^,^^3^ However, certain scenarios present challenges in determining the appropriate surgical procedure. These include instances of multiple lung cancer, lung metastasis, noninvasive lung cancer, or nonmalignant diseases located in areas unsuitable for wedge resection.^2^^,^^4^ Herein, we report on a case of multiple ground-glass opacities (GGOs) where a preceding right upper lobectomy facilitated the execution of a right middle segmentectomy, owing to the expanded volume of the residual lung.

The patient was an 81-year-old ex-smoking (20 pack-years) woman with a history of multiple primary lung cancer. She underwent left S4 and basal segmentectomy for lung adenocarcinoma pT1aN0M0 stage IA1 and pT2aN1M0 stage IIB at the age of 76 years. At the age of 77 years, she underwent robotic portal right upper lobectomy for triple lung cancer (pT1cN0M0 stageIA3, pT1aN0M0 stageIA1, and pT1aN0M0 stageIA1). Semiannual high-resolution computed tomography scans revealed a 14-mm part solid nodule (solid component, 10 mm) in S4a in right middle lobe with increased tumor size and density (Figure 1A). A standard uptake value max 2.6 was observed on positron emission tomography-computed tomography, indicating an accumulation of fluorodeoxyglucose, 2-[fluorine-18]fluoro-1-deoxy-D-glucose. Analysis of 3-dimensional computed tomographic images revealed an increase in the volume of the right middle lobe following right upper lobectomy: 293 mL before the right upper lobectomy and 360 mL after the right upper lobectomy (8% and 10.2% of total lung volume, respectively) (Figure 1B). Additionally, preoperative simulation confirmed that segmentectomy would ensure a surgical margin of larger than 1 cm. Consequently, a segmentectomy was performed to preserve the lung volume.

**Figure 1:**
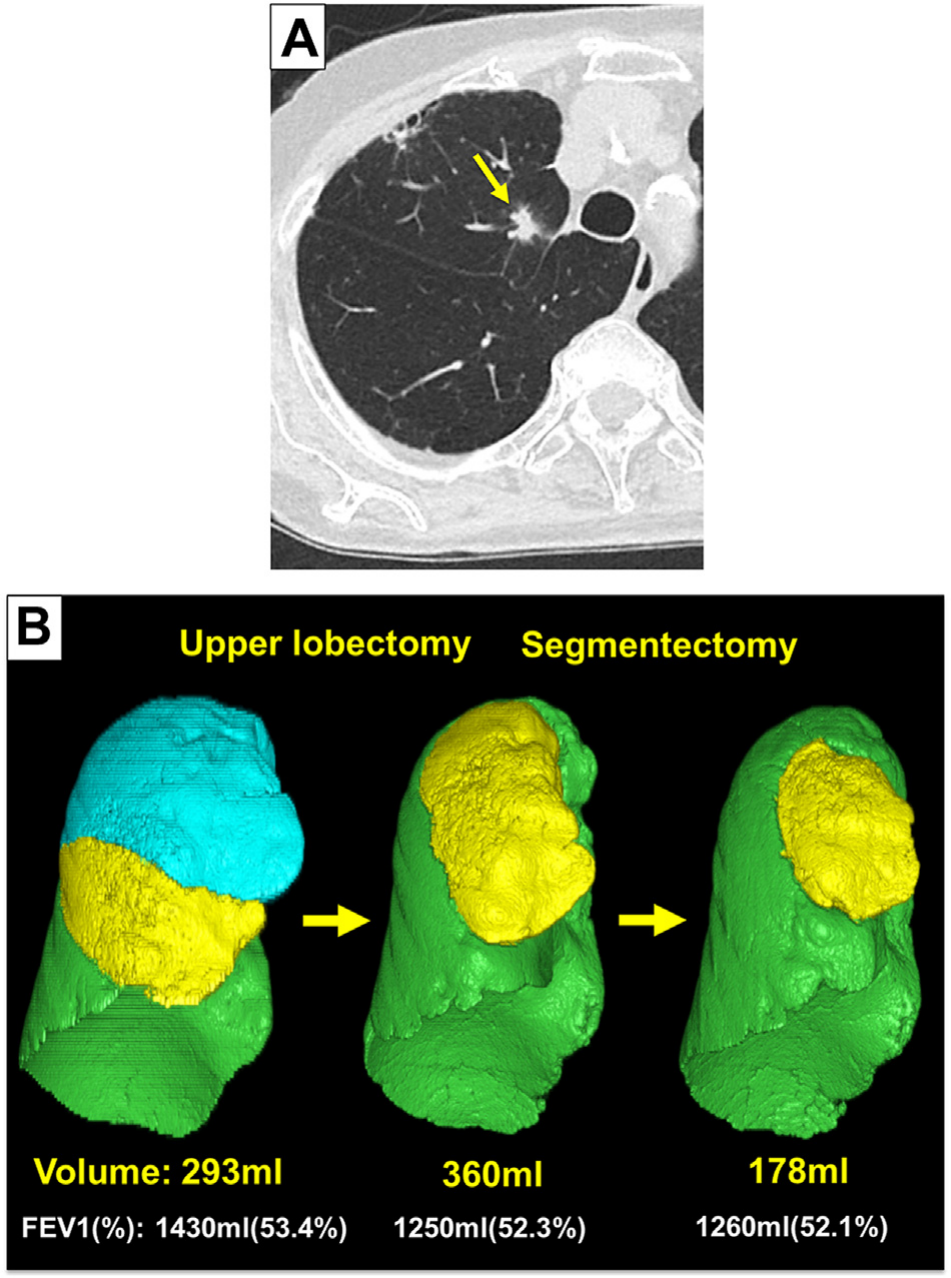
Representative computed tomography (A) and three-dimensional computed tomography images from lateral views before right upper lobectomy, after right upper lobectomy, and after right S4 segmentectomy (B). (A) Yellow arrow showing a 14-mm part solid nodule (solid component: 10 mm) in S4a in right middle lobe. (B) Lower label showing the volume of the right middle lobe and forced expiratory volume in one second (FEV1). Computed tomographic images were transferred and analyzed in three dimensions, and the volume of each lobe was calculated using Synapse Vincent (Fuji Film Co., Ltd., Tokyo, Japan).

Robotic portal surgery was performed under general anesthesia and epidural anesthesia, with the patient placed in a decubitus position (Supplemental video). Despite the presence of adhesions between the chest wall and the middle lobe resulting from a previous surgery, they were successfully removed. Subsequently, the interlobar fissure between the right middle lobe and lower lobe was divided using electrocautery. We identified A4 and A5a and dissected A4 using a surgical stapler (Figure 2A). V4a was dissected using electrocautery, and the target bronchus (B4) was dissected using a surgical stapler (Figure 2B). To guarantee a sufficient surgical margin, A5a was dissected using a surgical stapler (Figure 2C). The intersegmental plane was identified using indocyanine green fluorescence, and the intersegmental plane between S4 and S5 was divided using surgical staplers (Figure 2D). Because sufficient surgical margin was secured and lymph node metastasis was not observed by frozen section intraoperatively, lobectomy was deemed unnecessary. The postoperative course was uneventful, and the patient was discharged 6 days after surgery. The tumor was diagnosed as adenocarcinoma (pT1aN0M0 stage IA1), and follow-up chest computed tomography performed 6 months after surgery showed the absence of recurrent tumors. Furthermore, the surgical procedure did not adversely affect respiratory function (forced expiratory volume in 1 second, 77.1%-79.2%; vital capacity, 104.3%-110.5%; carbon monoxide diffusing capacity, 64.0%-59.3%).

**Figure 2:**
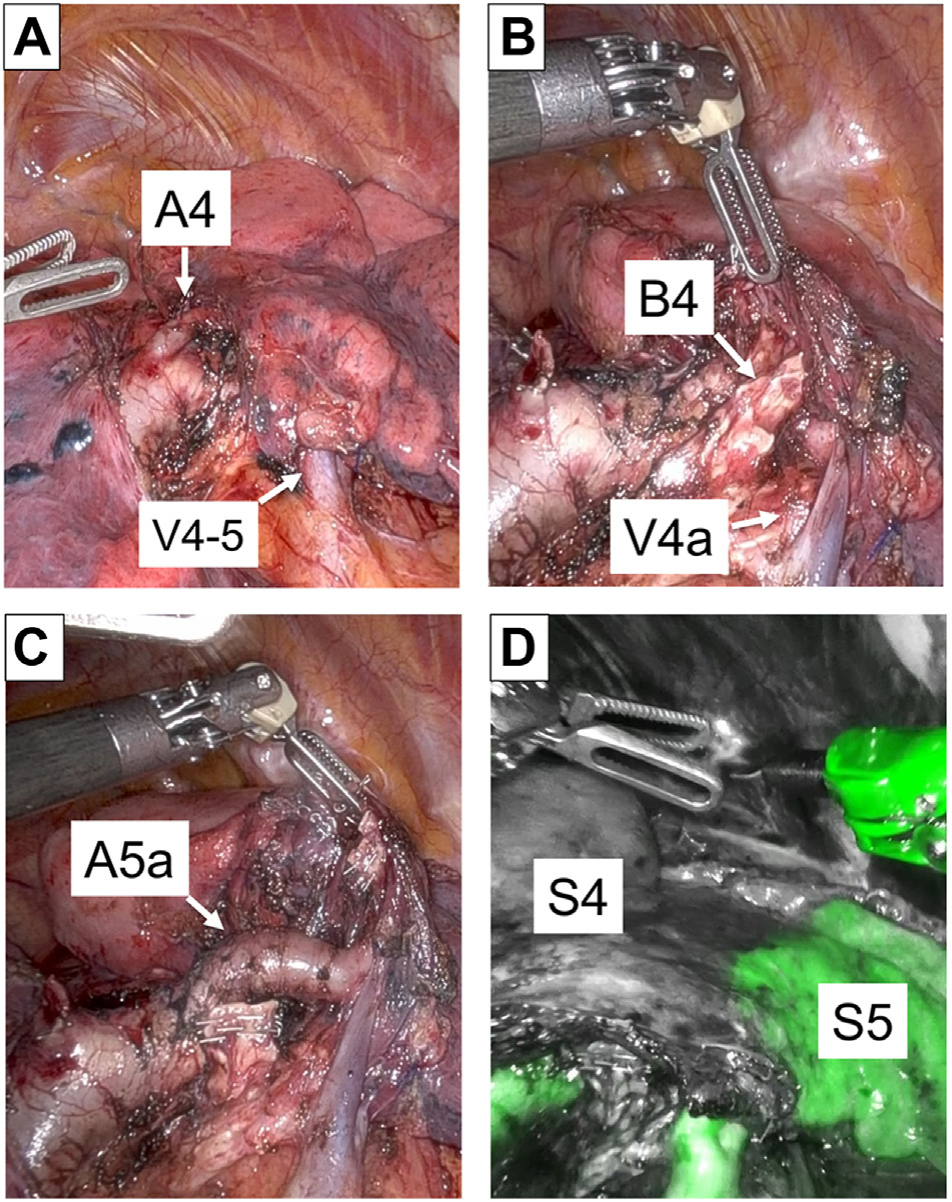
Intraoperative images showing (A) exposure of the A4 and veins, (B) exposure of the B4 and V4a, (C) exposure of the A5a, and (D) the intersegmental plane between S4 and S5 by indocyanine green.

## Comment

Although surgical resection stands as the primary treatment for lung adenocarcinoma with GGO component, the treatment strategy for multiple GGO lesions has not been established.^5^ Surgical resections should be considered in patients with suspected malignant subsolid nodules, easily accessible ipsilateral pure GGOs, or contralateral GGOs with increasing size or solid components during the follow-up period. The surgical strategy was determined based on the size, location, and computed tomography features of tumors, as well as on the performance status and pulmonary function of the patient. Because patients with multiple GGOs may necessitate multiple surgeries to achieve complete resections, the preservation of lung volume is essential in each resection. In the present case, the patient had previously undergone right upper lobectomy, left S4 segmentectomy, and left basal segmentectomy to remove 5 lesions. Therefore, the preservation of the remaining lung was of paramount importance.

The right middle lobe has the smallest volume among the lung lobes. Lobectomy is routinely performed to treat lung diseases affecting the right middle lobe, particularly in cases where lesions are located on the proximal side. Because the right middle lobe only accounts for 10% of the total lung volume, the execution of the right middle segmentectomy might not be commensurate with the expected benefits. Limited previous studies reported that the indications for right middle segmentectomy include lung metastasis, noninvasive lung cancer, and nonmalignant diseases located in areas unsuitable for wedge resection.^6^^,^^7^ Yajima and associates^2^ reported the case of middle lobe segmentectomy in patients with relatively large middle lobe. They emphasized that the right middle lobe volume should exceed 10% of the total lung volume. In the present case, although the right middle lobe was relatively small, it expanded from 293 mL to 360 mL (8%-10.2% of the total lung volume) after the right upper lobectomy. Hence, the performance of segmentectomy was significantly advantageous, given the resultant increase in the total lung volume. Several studies have examined the morphologic changes after lung resection through a volumetric analysis.^8^ It is imperative to conduct volumetric analyses when contemplating a second lung resection.

However, careful consideration is warranted when selecting this technique. Torsion and perfusion disorders in the remaining middle lobe postsegmentectomy are critical issues. These complications should be carefully monitored, and preparedness for potential completion of lobectomy should be maintained.

In conclusion, we experienced a case of a patient who underwent robotic portal segmentectomy in the right middle lobe. Previous right upper lobectomy caused the expansion of the middle lobe, which enabled us to perform segmentectomy. In patients with a naturally large right middle lobe or in patients with a relatively large right middle lobe due to previous lung resection, segmentectomy is a valuable option for preserving the lung volume.
